# Synthesis and Modification of Clinoptilolite

**DOI:** 10.3390/molecules22071107

**Published:** 2017-07-04

**Authors:** Pavlina Ambrozova, Jindrich Kynicky, Tomas Urubek, Vinh Dinh Nguyen

**Affiliations:** 1 Faculty of Forestry and Wood Technology, Mendel University in Brno, Zemedelska 1, 61300 Brno, Czech Republic; pavlina.ambroz@gmail.com (P.A.); urubek.tomas@seznam.cz (T.U.); 2 Central European Institute of Technology, Brno University of Technology, Purkynova 656/123, 61200 Brno, Czech Republic; nguyenvinhkhtn@gmail.com; 3 Faculty of Chemistry, Thai Nguyen University of Sciences, Tan Thinh Ward, 251580 Thai Nguyen, Vietnam

**Keywords:** zeolite, clinoptilolite crystallization, hydrothermal synthesis, HDTMA, surfactant modification

## Abstract

Clinoptilolite is a natural mineral with exceptional physical characteristics resulting from its special crystal structure, mainstreamed into a large zeolite group called heulandites. An overall view of the research related to the synthesis, modification and application of synthetic clinoptilolite is presented. A single phase of clinoptilolite can be hydrothermally synthesized for 1–10 days in an autoclave from various silica, alumina, and alkali sources with initial Si/Al ratio from 3.0 to 5.0 at a temperature range from 120 to 195 °C. Crystallization rate and crystallinity of clinoptilolite can be improved by seeding. The modification of clinoptilolite has received noticeable attention from the research community, since modified forms have specific properties and therefore their area of application has been broadening. This paper provides a review of the use of organic compounds such as quarter alkyl ammonium, polymer, amine and inorganic species used in the modification process, discusses the processes and mechanisms of clinoptilolite modification, and identifies research gaps and new perspectives.

## 1. Introduction

Clinoptilolite series minerals are the most common zeolites in nature and have been found in many areas all around the world, for instance, in Europe (Hungary, Italy, Romania, Slovakia, Slovenia, Turkey, former Yugoslavia), in Russia and several states of the former Soviet Union (Georgia, Ukraine, Azerbaijan), Asia (China, Iran, Japan, Korea), Africa (South Africa), Australia and New Zealand, and in many countries of the Americas, such as Argentina, Cuba, Mexico and the United States. Parent rocks commonly contain over 50% of clinoptilolite, but contents over 80% are very widespread too [1].

Clinoptilolite belongs to the group heulandite (HEU), which possesses a two-dimensional structure [2]. HEU tetrahedral framework is formed from tetrahedral SiO_4_ and AlO_4_ units and contains three sets of intersecting channels. Two of the channels are parallel to the *c*-axis: A channels are formed by strongly compressed ten-membered rings (aperture 3.1 × 7.6 Å) and B channels are confined by eight-membered rings (aperture 3.6 × 4.6 Å). C channels are parallel to the *a*-axis and they are also formed by eight-membered rings (aperture 2.6 × 4.7 Å). Clinoptilolite unit cells are monoclinic with space group C2/m [3,4,5]. The general chemical formula is (Na,K)_6_Al_6_Si_30_O_72_·20H_2_O [5,6] and the Si/Al ratio of clinoptilolite may vary from 4.0 to 5.3 [7].

Noteworthy properties of clinoptilolite are strong adsorption, high ion exchange and excellent molecular sieving [8]. These properties have been largely applied in many fields including agriculture, environment protection, pharmacy, petroleum technology, and construction [1]. In agriculture and horticulture, clinoptilolite has been used as a slow releasing carrier of fertilizers, insecticides, pesticides, antibacterial agents, and growth stimulators [9]. This kind of material is frequently used in environmental technology to remove heavy metals [10,11], dyes, and surfactants [12] from water or to eliminate toxic gasses [13]. Moreover, clinoptilolite can be used for producing antidiarrheal [14] and antiviral drugs [8], anticancer therapy [15], and drug carriers [16,17].

Although the mining of clinoptilolite from natural resources has been popular for many years, its artificial creation has been investigated in the last decades by many scientists due to the demand for high quality and purity of clinoptilolite. Consequently, there is a significant number of studies focused on the synthesis of clinoptilolite. Literature [18,19,20,21,22,23,24,25,26,27,28,29,30,31,32] demonstrates the efforts to synthesize from various sources of chemicals and different techniques to obtain clinoptilolite with different compositions.

Clinoptilolite is used not only in its initial form but also in its modified form. In recent years, numerous studies [15,17,32,33,34,35,36,37,38,39,40,41,42,43,44,45,46,47,48,49,50] have contributed to the issue of clinoptilolite modification. It was pointed out that the area of application could be intensively expanded by the functionalization of external as well as internal surfaces of clinoptilolite. The modification can be conducted by various chemical reagents such as surfactants, organic compounds [33,34,35,36,37,38,39,40,41,42,43], and inorganic substances [51,52,53,54,55,56,57,58,59,60,61,62]. The modification process can be single steps like cation exchanging [51,61] or multiple steps including cation exchanging, oxidizing, or reducing [55,58,59].

This paper presents details of the approaches that have been developed to synthesize clinoptilolite. Attention is given to the crystallization condition including starting materials, temperature, time, and amount of seeds. The production process is described in detail and a brief review of the strategies that have been established to modify clinoptilolite by various reagents is given. Furthermore, the applications of initial and modified forms of clinoptilolite are discussed.

## 2. Synthesis of Clinoptilolite

The synthesis of clinoptilolite can be carried out under a hydrothermal condition in an autoclave and controlled by some vital factors such as the composition of raw materials, reaction temperature, reaction time, and seeding as shown in Table 1.

### 2.1. Composition of Raw Materials

The raw materials used in synthesizing of clinoptilolite include silica, alumina, and alkali sources. Silica source may be colloidal silica [19,21,24,26,28,29,30], silica gel [18,20], fumed silica [24], and amorphous silica [24], from among in which colloidal silica is the most frequently used. The influence of silica sources on the formation of clinoptilolite has not much been in question. In the contribution reported by Williams [24], the authors supposed that the colloidal silica stabilized by ammonium ion was expedient for the synthesis of clinoptilolite. The raw materials providing alumina are varied. They can be aluminum hydroxide [18,21,23,27,28,29,30,32], sodium aluminate [20,22,23,29,30], aluminum [24,29,30], and aluminum salts [19,22]. The use of aluminum hydroxide was the most common way to produce pure clinoptilolite that could be obtained, while the use of aluminum salts was not preferred because it produced impurities. The most commonly used alkali source was a hydroxide form including sodium and potassium hydroxides. The alkali source could contain single or multiple alkali elements.

The vital role of the composition of the raw material with the formation of clinoptilolite was dealt with in many contributions. Hawkins [19] indicated that clinoptilolite could be synthesized from the raw material composed of MO:Al_2_O_3_:*x*SiO_2_, where MO could be CaO, SrO, or BaO and *x* was from 4 to 10. Goto [20] set up two experiment series with the raw material composition of Na_(2−*x*)_Al_(2−*x*)_Si_7+*x*_O_18_ and (Na,K)_(2−*x*)_Al_(2−*x*)_Si_7+*x*_O_18_, where *x* was 0.0, 0.5, and 1, and found that clinoptilolite only occurred in the presence of both K and Na when *x* was 0. One of the most important contributions to the synthesis of clinoptilolite was done by Chi et al. [21]. The authors indicated that a single phase of clinoptilolite could be obtained from the starting material with the composition of 2.1MOH:1Al(OH)_3_:5SiO_2_:52.5H_2_O, where M was Na or K. In contrast, other phases such as mordenite, phillipsite occurred with clinoptilolite when the raw material contained the mixture of Na and K or had other ratios. Kinetics study indicated that kinetics of crystallization of Na-clinoptilolite was found to be two or four times faster than K-clinoptilolite.

In order to investigate the synthesis of pure clinoptilolite in a mixed system of Na and K, Satoka et al. [23] used raw materials with a wide composition range and found that to obtain the clinoptilolite phase, the raw material mixture has to have the following composition in the molar ratio: SiO_2_/Al_2_O_3_ = 8–20; OH/SiO_2_ = 0.25–0.50; K/(K + Na) = 0.2–0.80; H_2_O/SiO_2_ = 10–100. In these, the combination of the molar ratio of OH/SiO_2_ and that of K/(K + Na) was particularly important. The influence of Si-Al ratio on the formation of clinoptilolite was disclosed by Zhao et al. (1997) and it was found that pure clinoptilolite could only be synthesized from gels having Si/Al ratios of 3.0–5.0 [25]. In the study of Zhao et al. (1998), the role of alkali-metal cations in the synthesis of clinoptilolite was investigated. Results showed that the nature of the alkali-metal cation did not have a critical structure-determining role in the synthesis but did contribute to other properties of the material including the rate of crystallization, the Si/Al ratio of the resulting crystals, the crystal size, and the morphology. Potassium ions greatly increased the rate of crystallization and decreased the nucleation time. Tanaka et al. [28] hydrothermally treated a (K,Na)-aluminosilicate slurry containing starting materials with Si/Al:(Na + K)/Si:K/(Na + K):H_2_O/Al molar ratio of 6.0:0.42:0.5:52.5 and found that plate-like particles of (K,Na)-clinoptilolite were formed. Guniver et al. [29] synthesized clinoptilolite according to the process reported by Chi et al. [21] and concluded that highly crystalline clinoptilolite could be obtained from the batch composition of 2.1Na_2_O:Al_2_O_3_:10Si_2_O:110H_2_O. In addition, clinoptilolite with good yield was obtained when Si/Al ratio was in a range from 5.0 to 6.0 [29]. When hydroxyl anions were partially replaced by chloride or carbonate ions, the crystallization rate was slowed down [30].

According to the above background, the ratio alkali:alumina:silica in the raw material is extremely significant for the synthesis of pure clinoptilolite. The most expedient ratio was described in the research of Chi et al. [21].

### 2.2. Synthesis Temperature

Generally, clinoptilolite was synthesized under a hydrothermal condition, so the reaction temperature played an important role. Before the study done by Chi et al. [21], researchers chose a very wide range of temperatures from 150 to 300 °C. However, the product often contained impurities [19,20] and the role of temperature was not clear. In order to investigate the crystallization kinetics of clinoptilolite, Chi et al. [21] prepared clinoptilolite at different temperatures, from 120 °C to 200 °C, and found that pure Na-clinoptilolite could be prepared at 120 °C, while mordenite occurred as a coexisting phase if the synthesis temperature was 140 °C. In contrast, pure K-clinoptilolite could be obtained at 195 °C, with impurities forming at a lower temperature. By using Arrhenius equation, the authors estimated the apparent activation energies for the crystallization of Na- and K-clinoptilolites. The values are 13.8 for Na-clinoptilolite and 14.5 kcal/mol which are close to that of synthetic mordenite, a coexisting phase with clinoptilolite in nature.

The dependence of the crystallization of Na-clinoptilolite on the temperature was investigated by Guvenir et al. [29]. The authors synthesized clinoptilolite at 100, 140 and 175 °C and revealed that at 100 °C, clinoptilolite with a crystallinity of 92% formed after 1918 h, while at 140 °C pure clinoptilolite was obtained after 118 h. On the other hand, mordenite crystallized at 175 °C.

It can be supposed that Na-clinoptilolite can be synthesized at a range of temperatures from 100 to 140 °C, while K-clinoptilolite is formed at a higher temperature—about 195 °C. When both Na and K are present in the reaction system, the temperature for synthesizing of pure (Na,K)-clinoptilolite is in the range from 140 to 180 °C, as shown in Table 1.

### 2.3. Seeding and Reaction Time

The role of seeding in the synthesis of clinoptilolite was firstly described in the study by Hawkins [19]. The author supposed that adding a few grains of clinoptilolite into the reactants was necessary for the reproducible synthesis of all the clinoptilolites, while in the absence of clinoptilolite seeds mordenite was the main product. In the contribution [21], pure clinoptilolite was obtained when clinoptilolite seeds were added into the starting material mixtures. This study concluded that the seed crystal improved both the crystallinity of clinoptilolite and the rate of crystallization. Satokokwa et al. [23] synthesized clinoptilolite with different levels of seeding and claimed that the crystallization time could be significantly decreased by adding clinoptilolite into the raw material mixture. The amount of the seeds should be 1% to 20% by weight based on the raw material mixture. The addition of the seed crystals may further support the framework formation reaction [23]. Tanaka et al. [28] studied the crystallization of clinoptilolite when 5% of natural clinoptilolite was added into the raw material and indicated that after 6 days the amorphous material remained essentially unchanged. However, after 8 days, the degree of crystallinity of clinoptilolite increased, while any amorphous material was no longer detectable. The role of seeding was evaluated in the contribution [29]. The author concluded that without seeds, clinoptilolite was not observed. In contrast, clinoptilolite was formed in all seeded samples and its crystallinity was in the range of 80–100% after 69 h. In conclusion, seeding is essential for supporting the crystallization of clinoptilolite.

Based on the literature, the period for synthesizing pure clinoptilolite varies from several hours to more than one thousand hours as shown in Table 1. However, the correlation between reaction time and the synthesis of clinoptilolite has not been fully elucidated and needs more investigation.

**Figure 1:**
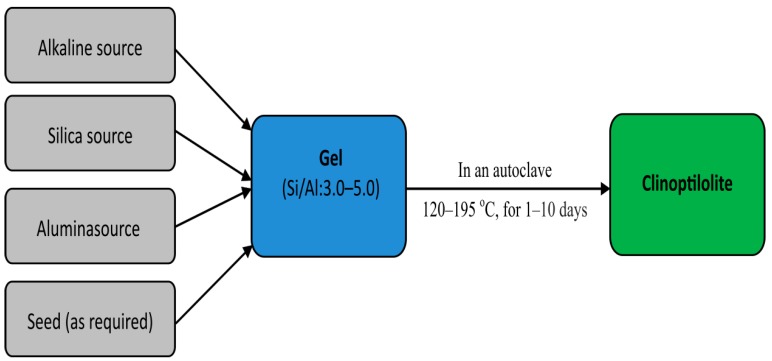
Schematic representation of the synthesis of clinoptilolite.

According to above analysis, the synthesis of clinoptilolite can be presented as shown in Figure 1. Initially, the sources of alkali, silica, alumina, and seed (as required) are thoroughly mixed to form a gel. Then, the gel is transferred into an autoclave in which the clinoptilolite will be crystallized under the hydrothermal condition. This process is governed by some key factors including temperature, seeding, Si-Al ratio in the starting material, and reaction time. Clinoptilolite can be synthesized at a temperature in a range from 120 to 195 °C, preferably from 140 to 150 °C. Seeding is necessary to improve the crystallization rate and can vary from 0 to 20% wt commonly 10%. The crystallization time depends on the amount of seeds, starting materials and can take from several hours to more than ten days.

## 3. Modification of Clinoptilolite

### 3.1. Organic Modification

Organically modified clinoptilolites are commonly produced by treating clinoptilolite with organic agents such as cationic surfactants [34,35,36,63,64], polymers [65,66], or amines [38,40,48,67]. The organic modification intensely changes the surface properties, allowing clinoptilolite to sorb anions or nonpolar molecules, for which the unmodified surface has a little affinity.

#### 3.1.1. Quarter Alkyl Ammonium Modification

Cationic surfactants have been used for modification of soil, clays, and zeolite since the 1980s and hexadecyltrimethylammonium ion (HDTMA) has been the most frequently studied. Boyd et al. [68] modified soils by using HDTMA for the removal of benzene and perchloroethylene. Results showed that the large alkyl ammonium ions were strongly sorbed onto soil surface, very difficultly displaced by smaller inorganic ions and the surfactant modified soil could effectively eliminate organic compounds from water.

Haggerty et al. [33] presented the modification of clinoptilolite by using HDTMA and revealed some important inferences. Namely, the interaction between surfactant and clinoptilolite is the ion exchange of HDTMA with extrastructural cations on the surface up to external cation exchange capacity (ECEC) of clinoptilolite. HDTMA-modified surface was stable when exposed to extremes in pH and ionic strength and organic solvents. Surfactant-modified clinoptilolite (SMC) effectively removed anions such as chromate, sulfate, and selenite, which were difficultly sorbed by unmodified clinoptilolite.

To elucidate the mechanism sorption of HDTMA onto the clinoptilolite surface, Sullivan et al. [34] used different initial concentrations of HDTMA (C_in,HDTMA_), including below and above critical micelle concentration (CMC), called monomer system and micelle system, respectively. For the monomer system, when sorbed below ECEC, HDTMA exists in the form of individual monomers on the surface of clinoptilolite, while if sorbed above ECEC, bilayer, patchy bilayer, or admicelles occur. For the micelle system, initially, HDTMA is taken up as an admicelle and exists in this form with the sorption above ECEC or rearranges to monolayers with the sorption below ECEC. The maximum uptake of HDTMA reached 160% and 250% of ECEC for the monomer and micelle systems, respectively. In addition, in the view of the energy of the system, the sorption of HDTMA onto clinoptilolite is the most stable for the micelle system with the sorption above ECEC.

Li et al. [35] investigated the sorption kinetics of HDTMA onto clinoptilolite by changing both surfactant and counterions concentration during sorption process. According to this study, the sorption is a function of mixing time and initial surfactant input (ISI). In agreement with the previous study [33], the results indicated that the HDTMA sorption onto clinoptilolite includes two stages. Firstly, HDTMA micelles were directly attached onto the clinoptilolite surface. Secondly, the surfactant on the surface rearranged, resulting in a transition from an admicelle to a monolayer when the ISI was less than the ECEC or a transition from an admicelle to an incomplete bilayer when the ISI was less than twice the ECEC. The time when surface rearrangement began depended on the ISI. Moreover, the counterion sorption/desorption data provided significant information regarding the surfactant surface configuration and, thus, should be included in studies of surfactant sorption. It is not sufficient to discuss surfactant sorption without counterion data [35].

**Figure 2:**
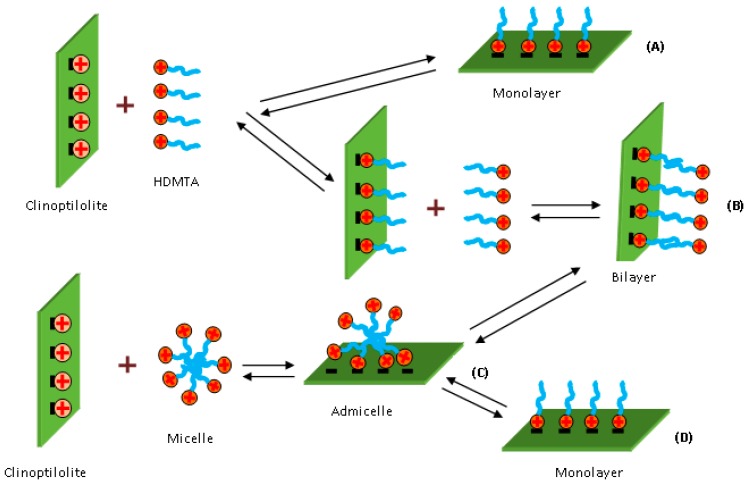
Conceptual model of hexadecyltrimethylammonium ion (HDMTA) adsorption onto natural clinoptilolite. (A) C_in,HDTMA_ < CMC and ISI < ECEC; (B) C_in,HDTMA_ < CMC and ISI > ECEC or C_in,HDTMA_ > CMC and ECEC < ISI < 2ECEC; (C) C_in,HDTMA_ > CMC and ISI > 2ECEC; (D) C_in,HDTMA_ > CMC and ISI < ECEC.

According to the above works, the adsorption mechanism of HDTMA onto clinoptilolite can be presented as in Figure 2. The surface properties of clinoptilolite have considerably changed after the modification. When C_in,HDTMA_ is below CMC, the HDMA monolayer (A) forms on the surface of clinoptilolite if ISI is under ECEC. While if ISI is above ECEC, initially the monolayer occurs, and then HDMA in the solution continues to interact with the first layer to form the bilayer (B). When C_in,HDTMA_ is above CMC, admicells (C) form on the surface if ISI is greater than 2ECEC. Admicells rearrange to form the bilayer (B) if ISI is in the range from ECEC to 2ECEC, and monolayer if ISI is below ECEC (D). In the case of the monolayer, the positively charged head of HDTMA is attached on the surface and the tail is oriented outwards, by which the modified clinoptilolite can take up polar and nonpolar molecules. When bilayer or admicells form, the positively charged heads are partly oriented outwards, which allows the modified material to be able to sorb anions such as chromate, selenate, phosphate, arsenate, and nitrate. These features have been used in many studies for various purposes. For instance, HDTMA modified clinoptilolite can be applied to environmental remediation [36,39,69], used to prepare slow-releasing fertilizers [32] or drug carriers [17].

Besides HDTMA, other quarter alkyl ammoniums such as octadecyldimethylbenzyl ammonium (ODMBA) [38], cetylpyridinium (CPD) [37,43,45], dioctadecyldimethyl ammonium [64] and tetraethyl ammonium [34] have been used to modify clinoptilolite. Aleksandra et al. investigated the adsorption of zearalenone, ochratoxin A and aflatoxin on natural clinoptilolite modified with octadecyldimethylbenzyl ammonium (ODMBA) ions and suggested that ODMA modified clinoptilolite only has a high affinity with the ionizable species [38]. Danina et al. studied the effectiveness of the CPD modified clinoptilolite on the release of diclofenac sodium in solution, and indicated that the modified material can prolong the release time of the drug [45].

#### 3.1.2. Polymer Modification

In recent years, the modification of clinoptilolite using materials other than only quarter alkyl ammonium has been investigated, and polymers including polyhexamethylene-guanidine [65,66,70], polyethylenimine [66], polyaniline [71], chitosan [72] and polypyrrole [73], with extremely useful characteristics such as multiple-functional groups or cross-linked forming, can combine with clinoptilolite to broaden the practical applications of clinoptilolite. Nikashina et al. [66] selected polyhexamethylene-guanidine and polyethylenimine containing amine groups for the modification of natural zeolite. Results showed that obtained materials have both the cation and anion exchange properties and acquire the bactericidal activity. In the research performed by Zaremotlagh et al. [71], modification of natural zeolite of clinoptilolite was done by the conducting polyaniline polymer. Modified clinoptilolite was applied to remove a dye from an aqueous solution and showed excellent adsorption capacity for the removal of methyl orange. In the work done by Zhao et al. [72], chitosan, a polysaccharide consisting of both amine and hydroxyl groups, was used to modify clinoptilolite. The thermodynamic parameters revealed that the sorption of Co(II) on chitosan–modified clinoptilolite was spontaneous and endothermic. The study showed that chitosan–clinoptilolite composite had excellent potential for the treatment of wastewater containing radiocobalt. Olad et al. [73] modified clinoptilolite by polymerization of polypyrrolein and outside of zeolite surface and found out that the prepared material could be used as aneconomic, and efficient adsorbent for Ni(II) ions removal.

#### 3.1.3. Amine Modification

Amines, with amino functional groups which can act as chelating species or form akyl ammonium ions, have also been used for the modification of clinoptilolite. Minchev et al. [67] investigated the interaction between n-octadecylamine, n-butylamine, tetrapropylamine and natural clinoptilolite. Wingenfelder et al. [40] treated natural clinoptilolite with cysteamine and propyl amine with the aim to remove Cd and Pb and found that amine-modified clinoptilolite could not be effectively used for this purpose. In the research performed by Guzel et al. [48], the interaction of natural clinoptilolite with primary amines including 1-dodecylamine, 1-hexadecylamine, and oleylamine was investigated. Results proved that the integration of amine groups was directly proportionate to the length of the hydrocarbon tail.

### 3.2. Inorganic Modification

In recent years, the modification of clinoptilolite by using metallic ions or oxides for specific purposes such as inhibition of bacteria, purification of water, and catalyzation of reactions has been investigated. Metals and their compounds with high activities like iron [53,54,56,57], aluminum [74,75,76], silver [59,60,61,62], titanium [77,78,79], copper [80], and manganese [54,55] were often utilized to modify clinoptilolite.

#### 3.2.1. Iron Compound Modification

Doušová et al. [52] treated clinoptilolite with FeSO_4_ solution at a room temperature (20 °C) for 24 h and found that the sorption capacity of Fe(II)-treated clinoptilolite increased significantly in comparison to the untreated material. During the modification process, the solid surface was laden with Fe(III) phases (hydrated oxides, ferrihydrite, FeOOH), which represented highly effective sorbents of arsenic oxyanions. In the same way, Guocheng et al. [57] prepared iron (II)-modified clinoptilolite for the removal of Cr(VI) from water and concluded that this material had a high affinity with chromate anions. Stanić et al. (2008) used a ferric chloride solution with different concentrations to modify clinoptilolite and indicated that iron uptake on zeolite increased with concentration. However, the iron compounds formed on the surface were not discussed. The estimated maximum of arsenic(V) adsorption to iron(III)-modified clinoptilolite based on Langmuir–Freundlich fit to the data was 1.55 mg/g [53]. Similarly, results performed by Šiljeg et al. [56] revealed the potential application of iron(III)-modified clinoptilolite for arsenic removal.

#### 3.2.2. Silver Compound Modification

Bogdanchikova et al. [58] modified natural clinoptilolite with a silver cation by reducing at a different temperature and found that Ag^+^ cations, Ag_n_ and Ag_n_^m+^ neutral and positively charged clusters, subcolloidal, and large Ag particles could exist in the prepared samples. All samples showed a significant microbicide action against *Escherichia coli* strain bacteria and turned out to be stable in air for more than three months. The natural clinoptilolite samples with Ag clusters and particles of different sizes were expected to be active in processes where a slow release of the Ag^+^ cation with olygodynamic properties is necessary. Likewise, Concepción-Rosabal [59] discovered the existence of Ag_2_^+^, Ag_4δ_^+^, Ag_80_, and Ag_8δ_^+^ clusters in the structure of silver modified clinoptilolite. De la Rosa-Gómez [60] treated natural clinoptilolite by sodium chloride, then by silver nitrate without reducing. Results indicated this kind of material could be used for the water disinfection processes. In the same way, but with the addition of pre-treating by oxalic acid or sodium hydroxide solutions, Copcia [61] found that the use of 2 mg Ag-clinoptilolite/mL medium of both samples as an antibacterial agent was adequate to stop the growth of *E. coli* and *S. aureus*. But if the amount was only 0.1 mg Ag-clinoptilolite/mL medium, the bactericidal action of silver-clinoptilolite pretreated with oxalic acid was more pronounced in comparison with silver-clinoptilolite pre-treated with sodium hydroxide, due to the different degree of silver exchange and to the differentiability of silver ions to move out of the crystalline network. Both antibacterial and heavy metal removal aspects of silver-modified clinoptilolite were investigated by Akhigbe et al. [62]. The results showed that silver-modified clinoptilolite could completely eliminate *E. coli* after 15 min and remove 97%, 98%, and 99% of Pb^2+^, Cd^2+^, and Zn^2+^ after 60 min, respectively. The amount of metal ions adsorbed by the zeolites in the single- and mixed-metal containing solutions was from 0.182 to 0.266 mg/g.

#### 3.2.3. Titanium Dioxide Modification

Titanium dioxide-modified clinoptilolite exhibited potential applications in the photo catalysis process. In the work reported by Nikazar et al. [78], TiO_2_-clinoptilolite was prepared by the solid state dispersion method in which TiO_2_ was mixed with clinoptilolite using ethanol as a solvent using agate pestle and mortar. Based on the results, this material had high efficiency in the photo degradation of azo dye Acid Red 114 in water. Similarly, but with the assistance of ultrasound, TiO_2_-clinoptilolite prepared by AkbariSene [81] had uniform active sites dispersion, high separation efficiency of electron–hole pairs and as a consequence, high surface density of active sites. Furthermore, this material showed sufficient reusability as optimal photo catalyst, making it a good choice for photocatalytic water splitting applications. Trujillo et al. [79] reported the preparation of TiO_2_-clinoptilolite using sol-gel method which includes dispersing clinoptilolite in a HCl solution, adding TiCl_4_ into the mixture, neutralizing by an NH_3_ solution, and heating at 100 °C. Anatase TiO_2_ was formed and the TiO_2_/clinoptilolite ratio of around 90/10 was found to be the most efficient in terms of a lower tendency to agglomeration and largest surface area. In the study of Yener et al. [82], TiO_2_-clinoptilolite was synthesized by the acid hydrolysis of TiCl_4_ on the clinoptilolite. The results showed the formation of the spherical rutile-TiO_2_ clusters was composed of nanofibers on the surface of the clinoptilolite and the dispersion of the TiO_2_ particles on the clinoptilolite led to a surface area larger than that of the bare TiO_2_ and clinoptilolite. The materials synthesized in the present study exhibited higher catalytic activity compared with the commercial Degussa P25 and anatase.

#### 3.2.4. Other Compound Modification

The inorganic modification of clinoptilolite is not only accomplished with the above-mentioned elements. Namely, in some studies [74,75,76], aluminum compounds were used, while some other researchers [55,80] employed copper and manganese compounds. Further, clinoptilolite can be modified by using a combination of different compounds, for instance, mixtures of Al^3+^, La^3+^ and ZrO^2+^ [75], of Fe^3+^ and Mn^2+^ [54], and of various ions [51].

## 4. Conclusions

The synthesis of clinoptilolite has been documented in many studies and it is still a big challenge to obtain pure clinoptilolite due to the lack of appropriate crystallization conditions. All of the studies have reached similar conclusions regarding the composition of the starting materials and temperature; however, further discussion is needed in order to determine the time needed for the crystallization. In addition, abundant possibilities have been observed in a prospective investigation of the role of stirring in the synthesis process.

It has also been concluded that the combination of clinoptilolite and organic compounds could have the potential to broaden the areas of application. The interaction between quarter alkyl ammonium ion, HDTMA, and clinoptilolite has been carefully investigated in some contributions. In contrast, the mechanism of modification by other organic compounds has not been well established and requires further study.

Clinoptilolite has ample versatility when it is combined with inorganic substances because of their variety of types and properties. Such combination brings some extremely useful characteristics for the composite material. Nevertheless, information regarding formation, such as the mechanisms and thermodynamics of clinoptilolite-based composite materials, has not been fully understood and needs to be further developed.

## Figures and Tables

**Table 1:** Experimental condition of clinoptilolite synthesis.

Raw Material Composition				Temperature (°C)	Time	Seeds (%)	Result	Reference
(Na,K)_2_Al_2_·Si_7_O_18_				200	65 days	0.0	Clinoptilolite Mordenite	[20]
2.1NaOH:Al(OH)_3_:5SiO_2_:52.5H_2_O				120	300 h	10.0	Na-clinoptilolite (100%)	[21]
				140	64 h	10.0	Clinoptilolite (90%) Mordenite (10%)	
2.1KOH:Al(OH)_3_:5SiO_2_:52.5H_2_O				175	94 h	1.0	Clinoptilolite (95%) K-feldspar (5%)	
				195	37 h	10.0	K-Clinoptilolite (100%)	
(2.1 ± 0.5)Na_2_O:Al_2_O_3_:(10 ± 2.0)SiO_2_:(110 ± 50)H_2_O				140	72 h	8.7	Clinoptilolite (56%)	[22]
				135–140	79 h	2.7	Clinoptilolite (67%)	
SiO_2_:Al_2_O_3_	OH:SiO_2_	K:(K + Na)	H_2_O:SiO_2_					
11	0.3	0.5	25	150	144 h	0.0	Clinoptilolite (100%)	[23]
11	0.3	0.7	20	180	24 h	1.0	Clinoptilolite (100%)	
10	0.3	0.6	20	150	72 h	10.0	Clinoptilolite (100%)	
0.72K_2_O:0.27Na_2_O:Al_2_O_3_:8.4SiO_2_:210H_2_O				150	336 h	0.0	Clinoptilolite (100%)	[24]
1.26Na:1.26K:Al:6.0Si:52.5H_2_O				140	8 days	5.0	Clinoptilolite (100%)	[28]
2.1Na_2_O:Al_2_O_3_:10Si_2_O:110H_2_O				140	118 h	10.0	Clinoptilolite (91%)	[29]
					141 h	3.1	Clinoptilolite (100%)	
					187 h	1.7	Clinoptilolite (100%)	
